# Evaluating the effect of extra-cerebral off-target binding in [F-18]MK6240 PET scans in early-stage Alzheimer’s disease

**DOI:** 10.1162/imag_a_00135

**Published:** 2024-04-18

**Authors:** Andrew McVea, Alexandra DiFilippo, Max J. McLachlan, Matthew D. Zammit, Barbara Bendlin, Sterling C. Johnson, Tobey J. Betthauser, Bradley T. Christian

**Affiliations:** Department of Medical Physics, University of Wisconsin – Madison School of Public Health, Madison, WI, United States; Waisman Laboratory for Brain Imaging and Behavior, Center University of Wisconsin – Madison School of Public Health, Madison, WI, United States; Geriatric Research Education and Clinical Center, William S. Middleton Memorial Veterans Hospital, Madison, WI, United States; Wisconsin Alzheimer’s Disease Research Center, University of Wisconsin-Madison School of Medicine and Public Health, Madison, WI, United States

**Keywords:** PET, Alzheimer’s disease, off-target binding, early detection, [F-18]MK6240, simulations

## Abstract

[F-18]MK6240 is a Positron Emission Tomography (PET) radioligand with favorable imaging characteristics for measuring tau aggregation in Alzheimer’s disease (AD). In this study, we investigated the impact of extra-cerebral off-target binding (ECB) in the meninges and sinus present in [F-18]MK6240 PET scans on quantifying tau burden in preclinical AD. Based on large cohort data from 433 [F-18]MK6240 scans acquired at the University of Wisconsin-Madison, simulations were conducted to examine the range of effects of ECB by varying the ECB profile and input radiotracer concentration curves on areas of early tau accumulation in AD. The range and patterning of ECB in the imaging cohort had high variability between participants; however, 35% revealed moderate to high meningeal signal that could influence quantification. Partial volume effects, which can lead to measured PET signal from neighboring regions influencing signal in adjacent areas of interest, were examined in the simulated images. The simulations demonstrate that signal from the sinus increases the neighboring entorhinal cortex region (ERC) signal and activity detected from the meninges can similarly influence the inferior cerebellar grey matter reference region. ECB effects from the sinus were the most prevalent in our cohort, and simulations with the average ECB profile had ERC uptake (SUV) 23% higher than simulations with no ECB. Spill-in effects from the sinus, which increases the medial and ventral temporal cortex standardized uptake value ratio (SUVR), and spill-in from the meninges into the cerebellar reference region, which leads to a reduction in global SUVR, act in opposite directions, complicating the interpretation of the derived SUVR of [F-18]MK6240 images. These simulation results quantify the effects of ECB in [F-18]MK6240 scans and introduce correction factors to minimize bias of the SUVR measure.

## 1 Introduction

Alzheimer’s Disease (AD) is the most common cause of dementia and an estimated 416 million people worldwide are living along the AD spectrum, including an estimated 32 million with AD-related dementia (Gustavsson et al., 2023). AD is characterized by the accumulation of beta-amyloid plaques and neurofibrillary tangles (NFTs) comprising aggregated tau protein. Physiologically, tau protein provides structure to neuronal cell microtubules; however, when hyperphosphorylated in AD, the tau aggregates into NFTs and accumulates within the neurons as AD neuropathology progresses (Goedart et al., 2017). The buildup of tau tangles is associated with neurodegenerative brain changes and cognitive impairment (Lowe et al., 2019). NFT aggregation typically follows a hierarchical pattern, described pathologically by Braak staging, starting in the transentorhinal cortex before emerging throughout the medial temporal lobe and cortex (Braak et al., 2006). As potential therapeutic treatments for AD are developed, accurate detection of tau at early stages before neuronal cell death and cognitive impairment is increasingly important for evaluating target engagement and treatment efficacy.

Due to its biomolecular specificity compared to other neuroimaging modalities and its ability to collect functional information*in vivo*, Positron Emission Tomography (PET) is a valuable tool for imaging and accurately quantifying the presence of AD-related pathological entities. [F-18]MK6240 is a PET radioligand that binds to AD-type tau aggregates with high affinity and can be used to noninvasively characterize NFT accumulation in preclinical AD (Hostetler et al., 2016). Compared to other tau tracers, [F-18]MK6240 has a higher selectivity for binding to tau versus background regions lacking tau pathology (Gogola et al., 2022;Hostetler et al., 2016). [F-18]MK6240 also demonstrates reduced off-target binding in regions such as the basal ganglia and choroid plexus, a common confounding factor with radiotracers including FDA approved flortaucipir (K. A. Johnson et al., 2016). Although off-target binding of [F-18]MK6240 in the substantia nigra (Gogola et al., 2022) has been reported, this is not a region used for quantifying NFT pathology. Outside of the cerebrum, [F-18]MK6240 has variable uptake in the meninges and sinus regions, particularly in the frontal, sphenoid, and ethmoid sinuses, surrounding the brain (Betthauser et al., 2019;Gogola et al., 2022;Mertens et al., 2022).

[F-18]MK6240 uptake in the meninges and sinus may lead to issues in PET quantification (e.g., standardized uptake value ratio (SUVR) measurement) due to the close proximity of these areas to cortical regions of interest for early detection of tau pathology. Due to the comparatively low spatial resolution of PET relative to the size of brain regions with early tau deposition, partial volume effects (PVEs) can influence the interpretation of reconstructed images. PVEs are typically manifested as a lower signal in high activity regions (with dimensions comparable to the resolution of the PET scanner) and in a smoothing effect, or reduced contrast, along edges between high and low signal regions. The influence of PVE is often most pronounced at the edges of the brain. For radiotracers with lower signal outside the brain, signals from regions on the edge of the cerebrum spread out into the lower uptake regions causing a spill-out effect. Conversely, in an image with higher signal outside the brain there can be increased cortical signal due to a spill-in effect. In [F-18]MK6240 images, the high extra-cerebral binding (ECB) signal from outside the brain can influence neighboring cortical regions, resulting in higher measured radiotracer concentration in these brain areas. The meninges are proximal to the outer layer of the cortex and adjacent to tau target regions, including the entorhinal cortex (ERC), and the inferior cerebellar grey matter, which is typically used as a reference region containing negligible tau-specific binding for PET analysis. Similarly, the sinus neighbors the mesial temporal lobe, including the ERC. Increasing sinus and meninges PET radioactivity concentration can potentially bias signal in the target regions used for AD quantification and influence how the PET images are interpreted. This is particularly problematic for imaging and identifying individuals with preclinical AD as tau burden in the medial temporal cortex is beginning to emerge, which is critical for defining the earliest presence of tau pathology and understanding its relationship with decline in cognitive function. The measurable elevation of [F-18]MK6240 binding can be masked by proximal off-target signal in this region. In cases with high ECB, early characterization of tau pathology will present a technical challenge as the development of novel therapies targeting amyloid and tau removal will require accurate assessment of changes in neuropathology.

The overall goal of this work is to further understand the impact of [F-18]MK6240 off-target signal and its effect on detecting tau neuropathology at the earliest stages of emergence with a specific focus on ECB in the meninges and sinus and its effects on the SUVR measure in the entorhinal cortex. This work will investigate: 1) the frequency and extent of ECB observed in [F-18]MK6240 PET scans from our ongoing research studies at our site; 2) develop and validate simulated PET scans that replicate the distribution of radiotracer observed in our human investigational scans; 3) characterize the effects of ECB spill-in from the meninges and sinus on the target regions of the ERC and insula (control region); 4) derive correction factors to restore accuracy of target-specific binding of [F-18]MK6240 (measured using SUVR) in the presence of ECB and compare with a commonly used method for partial volume correction; and 5) assess the sensitivity of [F-18]MK6240 PET scanning for detecting tau aggregates in in the presence of ECB.

## 2 Methods

### 2.1 Study participants and PET scanning procedures

Participants from studies as part of the Wisconsin Alzheimer’s Disease Research Center (ADRC) or the Wisconsin Registry for Alzheimer’s Prevention (WRAP) (S. C. Johnson et al., 2018;Sager et al., 2005) who underwent [F-18]MK6240 PET imaging at 70-90 minutes post-injection on a Siemens ECAT HR+ were included in the study. In total, 433 [F-18]MK-6240 scans from 300 participants with a nominal injected dose of 10 mCi (370 MBq) from dates ranging from June 2017 to April 2022 were included in these analyses. Written consent was obtained prior to any participant procedures according to the Declaration of Helsinki.

All [F-18]MK-6240 scans were processed using an in-house processing pipeline (Betthauser et al., 2020). The four 5-minute PET frames were reconstructed using a ramp filter (128x128x63, 2.574x2.574x2.425 mm^3^voxel size) and then processed using a standardized MATLAB processing pipeline in SPM12 (Ashburner et al., 2011). PET images were first smoothed using a 6x6x6 mm^3^gaussian kernel; then, individual frames were aligned and summed. The summed image was coregistered to the participant MRI and then converted into an SUVR image using the inferior cerebellar grey matter ROI from the Medical Image Computing and Computer Assisted Interventions (MICCAI) atlas as a reference region. For additional comparisons between participants, the images were then spatially normalized into MNI-152 space (182x218x182, 1x1x1 mm^3^voxel size) by applying an affine transform between skull-stripped participant MRI and MNI-152 template brain. This analysis pipeline was previously used to extract time activity curves and calculate SUVR from regions in the Harvard-Oxford atlas (HOA) (Desikan et al., 2006;Frazier et al., 2005;Goldstein et al., 2007;Makris et al., 2006) in [F-18]MK6240 studies as outlined inBetthauser et al. (2020).

### 2.2 [F-18]MK6240 distribution characterization

To evaluate ECB in our imaging population, the SUV and SUVR of the meninges and sinus were quantified using custom ROIs. Following the method outlined inSmith et al. (2021), a meninges ROI was created for analysis by dilating the MNI-152 cortical brain mask by 5 mm in MATLAB. The original brain mask was then subtracted from this dilated mask to create a 5 mm-wide ROI circumscribing the outside of the cerebrum. Using an averaged image of our 433 [F-18]MK6240 scans in MNI space, the sinus—including the frontal, sphenoid, and ethmoid sinuses where elevated signal is seen in [F-18]MK6240 images—was segmented using ITK-SNAP following the anatomical borders of the MRI while also including the major areas of elevated binding seen in the average image around the sinus.

**Fig. 1.**
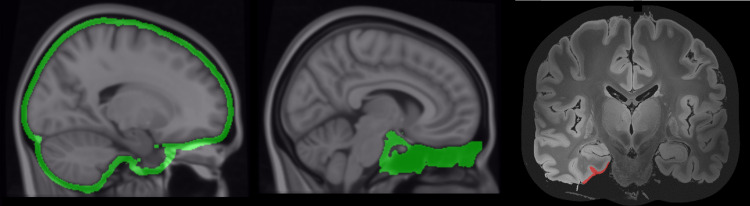
Custom ROI for meningeal (left), and sinus (center) uptake used for our analysis overlaid on the MNI-152 brain template. The ROI for the ERC (right) was segmented on the ultra-high-resolution MRI shown (discussed inSection 2.4).

**Fig. 2.**
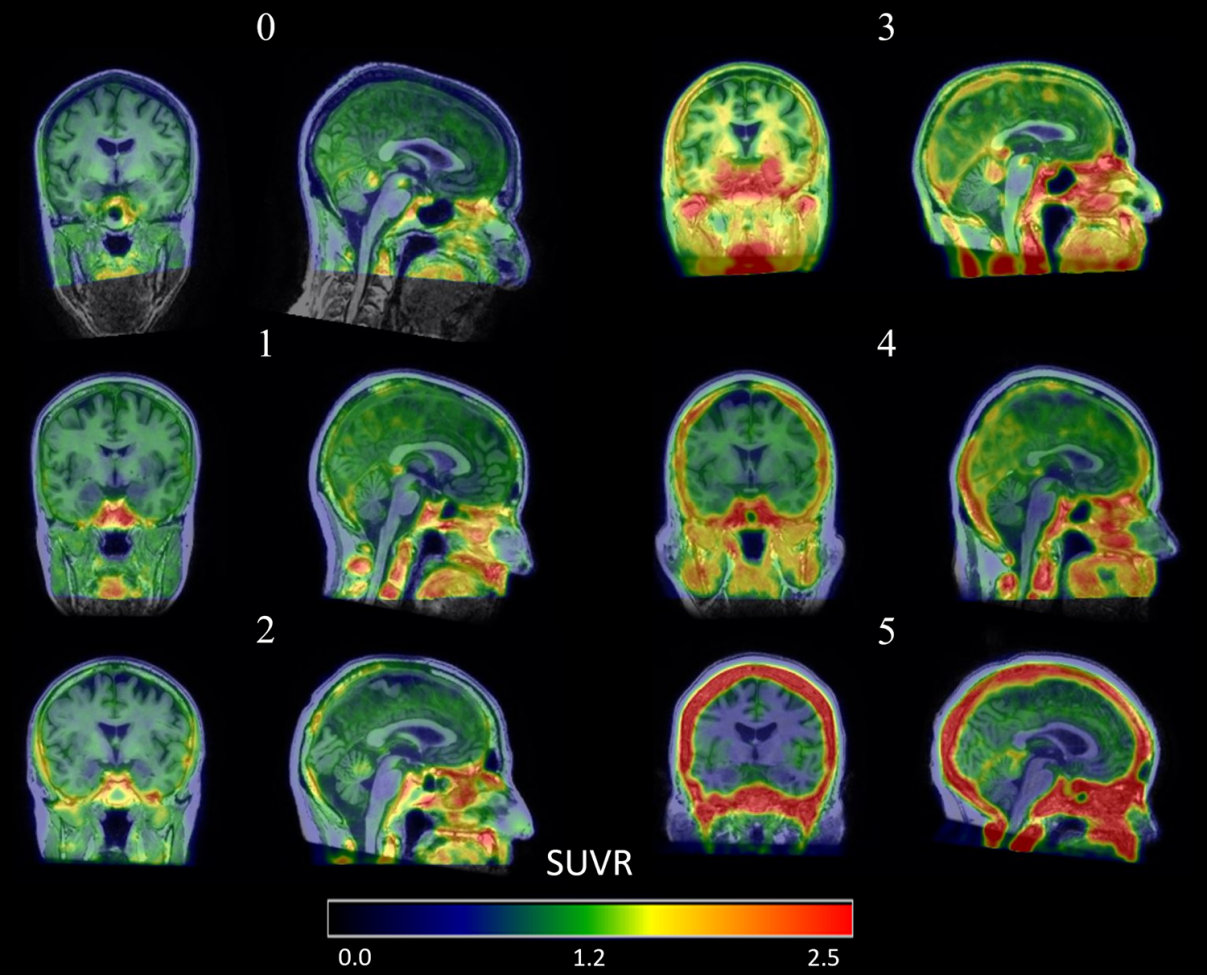
Illustrations of ECB in [F-18]MK6240 images acquired at our site showing the visual rating system (0–5) for meningeal signal. Note the increasing magnitude and frequency of hot spots of signal around the cortex with increasing visual rating, particularly near the occipital edge of the cortex in these examples.

The meninges and sinus ROIs used for quantification (Fig. 1) were then applied to all [F-18]MK6240 PET scans using SUVR as a metric of specific binding in these two regions in MNI space. As a part of our QC process, the [F-18]MK6240 images were rated with a score from 0–5 qualitatively ranking meningeal signal in relation to evaluating PET measures by a trained neuroimaging data scientist using a standardized QC manual. A score of 0 indicates little to no meningeal signal, 1 has one or two hot spots but no influence on cortical regions, 2 has multiple focal hot spots and limited cortical influence, 3 has signal spilling into the cortex but in localized regions, 4 has more intense signal spill-in and over a greater area, and a score of 5 represents high signal spill-in from the meninges with a strong concern for influence on quantification. Sample images from each of these scores (Fig. 2) show a steady increase in ECB in both the meninges and sinus, as well as the increased presence of local signal hot spots. The QC scores are primarily focused on signal from the meninges; however, there is an overlap between the meninges and sinus ROI and high sinus binding often influences the rating due to being a signal hot spot (scores >1) or having signal spilling into the cortex (score >3). Using these scores as a guide, we stratified our imaging data into three tiers using receiver operator characteristic (ROC) curve analysis based on the range of meningeal SUVR seen in these rated PET scans: Low (a score of 0–1 with little to no influence of ECB), Moderate (score of 2–3 with some influence), and High (score of 4–5 with strong influence).

### 2.3 Simulation tool

Simulations were performed using the PET-SORTEO package (Reilhac et al., 2004,^2005^), which is a Monte-Carlo based simulation software. SORTEO uses a voxelized emission map consisting of a digital brain phantom with binary brain regions and corresponding input radiotracer concentration curves (RTCCs), which plot the concentration of a PET ligand in a region over time to generate a realistic PET image based upon the*in vivo*(i.e., phantom) distribution of a radiotracer and the physical characteristics of the specific PET scanner model. Along with the emission map, an attenuation map differentiating tissue classes is input to SORTEO to model photon attenuation. The simulations account for sources of noise from the detector and random physical properties while keeping variables such as anatomy, metabolism of the radiotracer, and binding distribution within the brain consistent as defined by the voxel RTCCs. This allows the ability to isolate the effects of ECB profiles and increasing concentration of tau in the ERC, while maintaining realistic PET scanner-specific imaging characteristics.

### 2.4 PET emission map for [F-18]MK6240

Using the HOA as the neuroanatomical reference, a digital brain phantom was created consisting of 89 regions with associated RTCCs. The HOA was chosen because of its alignment with temporal cortex ROIs used in Braak staging as currently used for ROI analysis at our site (Betthauser et al., 2020;Desikan et al., 2006;Frazier et al., 2005;Goldstein et al., 2007;Makris et al., 2006). The unedited HOA comprised 115 brain regions primarily consisting of cortical grey matter with separate left-right segmentations in all regions except for the brain stem. To simplify our model and reduce processing time, left and right regions were combined leaving a total of 58 HOA regions.

The Automated Anatomical Labeling (AAL) atlas (Tzourio-Mazoyer et al., 2002) served to model the cerebellar grey matter and vermis, providing greater detail than the HOA, resulting in 17 cerebellar regions from the AAL atlas into the emission map. The snake tool in ITK-SNAP was used to segment remaining interior brain regions that were not already specified by the HOA into general white matter and ventricle ROIs based on the MNI-152 T1-weighted template MRI. Due to the known off-target binding of [F-18]MK6240 in the substantia nigra, this region was also included in the simulations based on the nigral organization atlas (Zhang et al., 2017).

Early tau deposition in AD generally follows a well-established pattern of progression as described by the Braak stages (Braak et al., 2006). Our investigation focused on the first stage of NFT accumulation in the ERC. Using published pathology in the Braak paper as a reference, the Braak I volume of tau accumulation was segmented by hand in ITK-SNAP on a 200µm ultra-high-resolution MRI in MNI space (Edlow et al., 2019). The individual layers of the brain tissue are visible in this MRI and allow for the segmentation and comparison of results to published tau pathology. This region of entorhinal tau accumulation lies within the anterior parahippocampal gyrus (APHC) as defined by the HOA (Fig. 1) and for this study the APHC is used for ERC analysis.

**Fig. 3.**
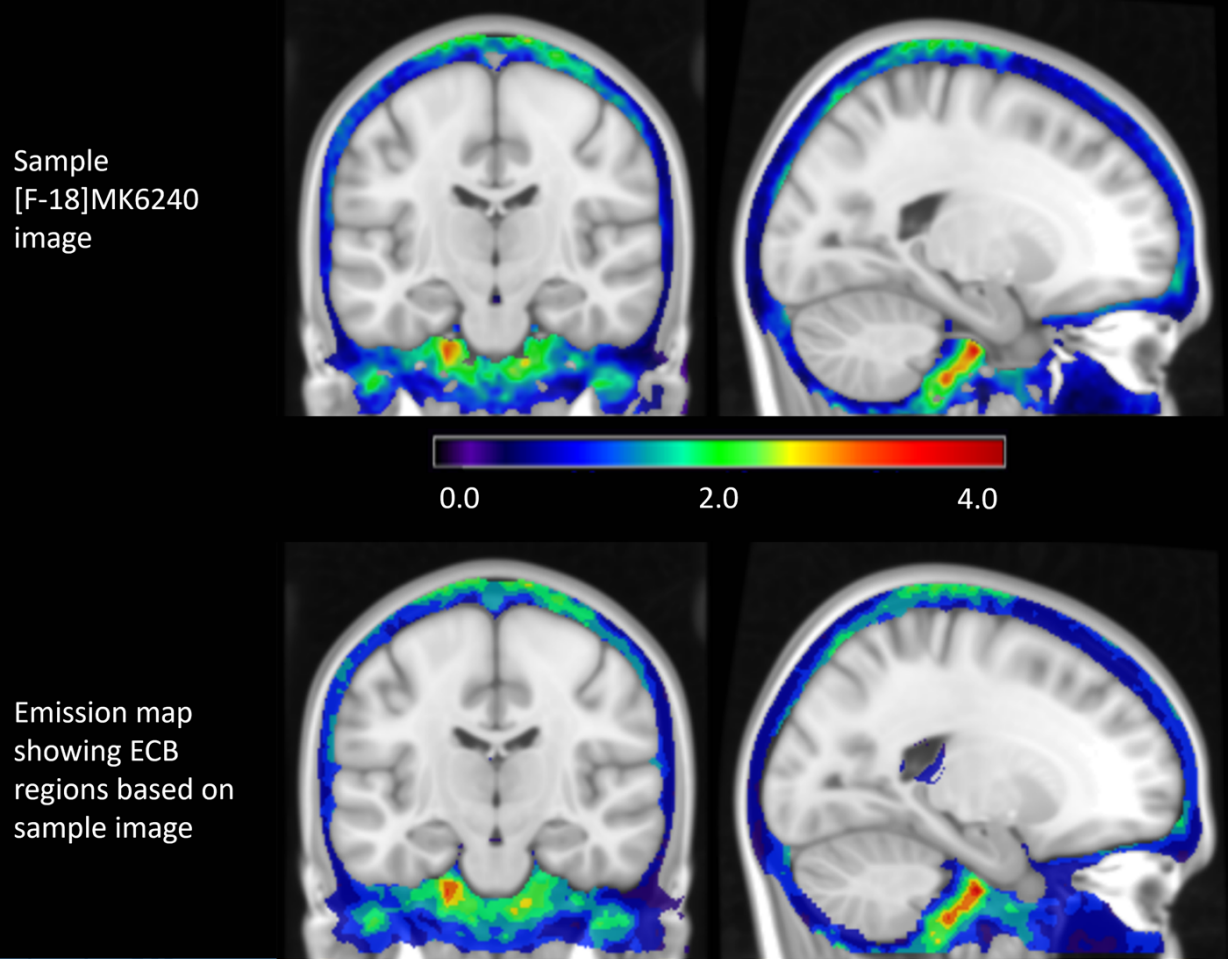
Coronal and sagittal slices showing an example [F-18]MK6240 ECB distribution (above) that became the basis of the HIGH simulation group. After binning the ECB by signal, the 10 resulting voxelized ECB regions (below) are added to the emission map for simulation.

Unsimulated PET images and time activity curves collected at our site were used as the basis for the RTCCs for the regions described in the emission map. To account for the varied ECB seen in participants, different off-target binding maps were created based on [F-18]MK6240 scans of our research cohorts. An ECB mask that encompassed the meninges, sinus, and nearby extra-cerebral areas was applied to these images and voxels in these regions were histogrammed according to measured PET signal into 11 bins. The first of these bins contained all voxels less than or equal to zero and were not included in the emission map. The voxels from the individual non-zero histogram bins were combined to create 10 discontinuous off-target binding regions based on the radioactivity concentration observed in human participant PET scans, resulting in a series of emission maps representing the distribution and magnitude of ECB (Fig. 3).

Using this method, six different emission maps were created with unique ECB profiles for comparison. The first was based on a participant with the lowest sinus and meninges SUVR from our analysis described inSection 2.2(LOW). A second and third emission map were then based on the ECB of the participant scans with the highest meningeal SUVR (H_MENINGES) and highest sinus SUVR (H_SINUS). While these are more extreme cases, an additional map with a meninges and sinus SUVR 60% above average (HIGH) was added to capture the full range of the effects of ECB within our dataset. These emission maps were based on individual [F-18]MK6240 scans in order to realistically represent the range of PET scans acquired at our site and to include hot spots of ECB signal seen throughout the image, which is not necessarily captured in an average image. Finally, we generated emission maps based on the average ECB profile of all participant scans (n = 433) (AVG) and an idealized simulation group with ECB set to a uniform background (BKG). In the BKG group, all regions outside the brain were given an RTCC equal to half of the inferior cerebellar RTCC, thus resulting in a theoretically uniform SUVR of 0.50 outside the cerebrum. This radioactivity concentration for the BKG group was selected to minimize the spill-in effect on the regions of interest, while also avoiding any spill-out effects that would artificially decrease the signal in target regions. A description of the six simulation groups is given inTable 1presented in order of increasing sinus signal, with the ECB regions visualized inFigure 4.

**Table 1.** ECB SUVR in simulation groups.

ECB group	SINUS (SUVR)	MENINGES (SUVR)
BKG	0.50	0.50
LOW	0.72	0.66
AVG	1.38	1.03
HIGH	1.53	1.38
H_MENINGES	2.01	2.10
H_SINUS	3.09	1.28

**Fig. 4.**
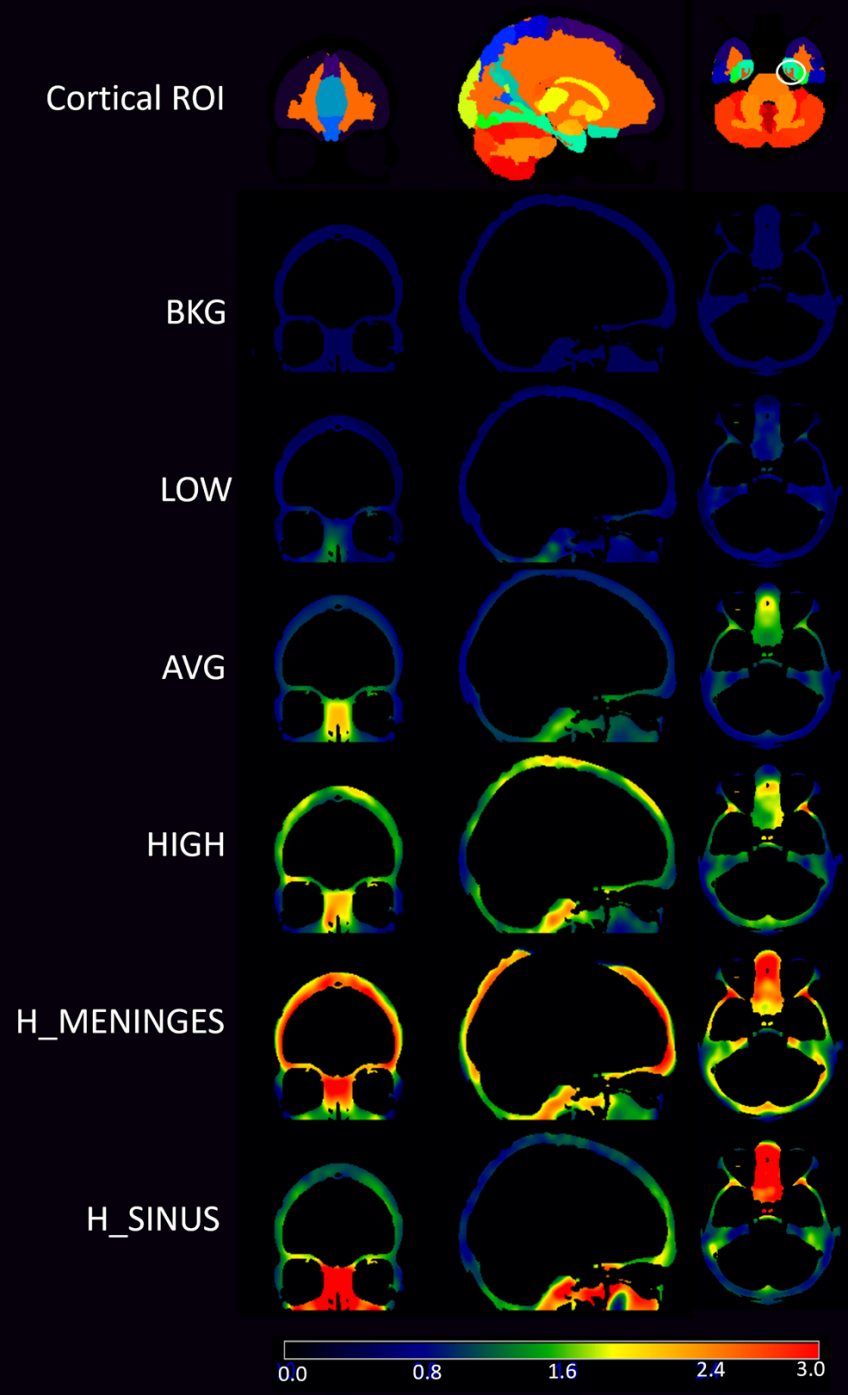
Components of the [F-18]MK6240 emission maps used to simulate different ECB patterns using SORTEO. The cortical ROI (top row) have identical RTCCs for each simulation cohort, including the Braak I region (circled in white). The distribution of the 10 discontinuous ECB regions and their corresponding RTCCs do differ between simulation cohorts, though, and are based on the distributions shown in the subsequent rows.

### 2.5 Simulations of [F-18]MK6240 PET

The SORTEO generated data were based on the Siemens CTI ECAT HR+ PET scanner and modeled after a 20-minute [F-18]MK6240 scan composed of 4 x 5 minute time frames starting 70 minutes post-injection of radiotracer. This PET scanner has FWHM = 4.1 mm in plane resolution and 4.3 mm radial and tangential resolution at the center of the field-of-view (Brix et al., 1997). An injected dose of 10 mCi (370 MBq) and a participant weight of 60 kg (132 lbs) was used for the simulated subject and unless otherwise specified, the default sensitivity and scanner settings were used. Simulations were multithreaded across 20 cores, requiring approximately 2.5 hours for each realization. A total of 10 realizations were simulated for each group. Reconstructions of our simulated images were completed using the same research protocols as participant scans using filtered back projection (zoom = 2) with an all-pass filter to a 128x128x63 matrix with voxel dimensions of 2.574x2.574x2.425 mm^3^. Unlike our human research studies, motion correction was not performed on the simulated frames before summing the frames across the four 5-minute PET frames. Additional smoothing was also not applied to the simulated images since the RTCCs were based upon radioactivity concentrations from already smoothed images and have a common spatial resolution with the participant images in standard space.

Using a rigid body transformation, the resulting PET images were then aligned with the MNI-152 standard space MRI for analysis. Note that since the simulations were based on emission maps set in MNI-152 space that standard space and MRI space are the same.

To validate the simulation tool, 100 realizations were run on the AVG binding group and the SUVR information was compared between the simulated data and the average of the participant data collected at our site. This high number of realizations was chosen to ensure stability of the simulations over time and to have a similar order of magnitude of scans as with the participant data. The average SUVR of these realizations in regions relevant to AD neuropathology were then calculated and compared to the ground truth values derived from the average SUVR in our imaging population to ensure that our simulations correctly represent the distribution of the observed [F-18]MK6240. Additionally, the average simulation image and the average image of all [F-18]MK6240 scans at our site were visually inspected for major differences using a subtraction image scaled to the SUVR of relevant ROIs in the HOA. Close scrutiny was given to the APHC as defined by the HOA, which contains the ERC, the inferior cerebellar grey matter reference region, hippocampus (HC), and the insular cortex (IC), which is isolated from the meninges and sinus in the cerebrum and is used as a control region unaffected by ECB.

### 2.6 Spill-in effects

The influence of radioactivity from the meninges and sinus spilling into the cerebellum and ERC, respectively, were quantified using SUV (=PET conc / 10 mCi * 60 kg) in order to isolate their effects. The use of SUV was chosen as an appropriate strategy for analyses of the simulations because the input radiotracer metabolism and kinetics were kept constant between realizations. However, the SUVR metric was used for comparisons involving real participant data. Participant SUV is highly variable, having a range in our cohort of 2.1 to 27.9 in the ERC among tau negative participants. Higher SUV values can be associated more closely with increased radiotracer delivery (representing flow and brain penetrance) of an individual than with higher radiotracer-specific binding in a target region. Normalization to a reference region in the calculation of SUVR accounts for these effects and for this reason, comparisons of human participants were completed using SUVR focused on the ERC and not SUV.

Using the BKG group simulations as a baseline with no specific radiotracer binding in the meninges and sinus, we observed the effect of increasing the meningeal and sinus signal along the patterns of the five additional simulation groups described inTable 1while keeping cerebral RTCCs constant between simulations. The inferior cerebellar grey matter is the most commonly used reference region for [F-18]MK6240 analysis; however, alternative reference regions that are more resilient to ECB effects have also been proposed. These include an eroded inferior cerebellar grey matter region (eroded by 3 mm), the pons (as defined by the Freesurfer v 7.1.1 segmentation), and the cerebral white matter (Freesurfer segmentation and 4 mm erosion) described using the methods inFu et al. (2023). The subsequent change in SUV of the proposed reference regions (with a focus on the commonly used inferior cerebellar grey matter) and ERC from different ECB patterns were compared to the increasing SUV of the meninges and sinus for these groups using the previously described ROIs.

### 2.7 Detection threshold of elevated [F-18]MK6240 binding

Along with examining the effects of ECB in tau negative scans, we also investigated how spill-in signal can bias the ability to detect low-level tau-specific binding of [F-18]MK6240 which is indicative of early tauopathy. This investigation focused on the changing radioactivity concentration of the radiotracer in the target brain regions and did not attempt to uncouple*in vivo*kinetics dependent on the binding (B_max_, K_D_) and nondisplaceable (V_ND_) signal of [F-18]MK6240 for tauopathies. To quantify the positive detection of PET signal, the [F-18]MK6240 threshold was defined for detecting elevated binding in the simulation group as the input time-activity concentration necessary for the simulated PET image to be classified as tau positive, allowing us to compare an index for pathology concentration with the derived PET images. Following the methods used inBetthauser et al. (2020), the PET threshold was set to be considered tau positive as an ERC SUVR of at least 1.27, using no corrections for PVE. To determine the corresponding input time radiotracer concentration of our simulations with different ECB groups, the magnitude of the radioactivity concentration of [F-18]MK6240 in the Braak I region was varied described above. A total of 10 realizations for each ECB profile were created at the different Braak I radioactivity concentrations until the average of those realizations had a mean value two standard deviations above the 1.27 threshold. This ensures that given the normal nature of the random processes in SORTEO >95% of scans imaged with this radioactivity concentration would be classified as tau positive. The corresponding input radioactivity concentrations were determined for each of the ECB profile groups included in this study as well as the SUV and SUVR of target ROI. Since the 1.27 SUVR threshold was determined based on the average signal across the population imaged with [F-18]MK6240 at our site, our comparisons of input activity detection threshold are driven by differences from the AVG simulation group.

### 2.8 Derivation of ECB correction factors

Along with identifying the extent of the problem that ECB can lead to in [F-18]MK6240 imaging, this study examined potential correction factors to account for spill-in effects to target and reference regions. A variety of published methods have been reported to correct, or recover, the degradation in PET due to partial volume effects (Yamao et al., 2018). This process works to decrease signal spillover from adjacent regions, particularly in areas with high contrast. Relevant to this investigation is to correct for sinus activity contaminating the ERC signal, and meninges activity contaminating the cerebellar reference region signal. The investigated method used grey and white matter probability maps from an MRI associated with the PET scan to correct for spill-out from grey matter and spill-in from white matter based on the underlying brain anatomy and the spatial resolution of the PET scanner. For this study the Muller-Gartner (M-G) method of PVC (Müller-Gärtner et al., 1992) using the same process described byMertens et al. (2022)was applied. Briefly, tissue probability maps were generated for GM, WM, and CSF in MNI-152 space using the segmentation tool in SPM12. Then, using a 6.5 mm point spread function and assuming a homogenous white matter uptake, the signal contributions from the white matter and ECB (represented by the CSF segmentation) were calculated for each voxel in the grey matter and subtracted to provide the corrected radiotracer concentration. While there are multiple PVC methods available, this study focused only on the M-G method due to its common use in PET imaging and wide availability. PVC was performed on the summed, simulated images created in MNI-152 MRI space.

A region-specific, linear correction factor was calculated using the relationship derived from the influence of meninges signal on the inferior cerebellar reference region and the sinus signal on the ERC. These simulation-based correction factors (SCFs) estimate the expected ECB contribution to the target ROI using the average spill-in signal determined across the ECB spectrum. These factors are then subtracted from the ROIs. To evaluate the SCFs, we then compared the accuracy of both SUV and SUVR with the PVC corrections using the simulated data. The SCFs were then applied to the participant data to evaluate the extent to which this correction removed any group-based differences between the tau negative ECB groups. Since ERC SUVR is one of the most commonly used target regions and spill-in primarily comes from the sinus, the participant data were partitioned into quartiles based on sinus SUVR. Then, a two-tailed t-test was used to compare the ERC SUVR in each quartile before and after correction to identify any significant differences between groups.

## 3 Results

### 3.1 Characterization of UW Research [F-18]MK6240 scans

Ages of the participants ranged from 27 to 93 years at the time of scan, although a majority of the participants were in their 60s and 70s with an average age of 68.7(7.3) years at the time of the [F-18]MK6240 PET scan. 67% of the participants were female with an average weight of 80.4(18.3) kg. A majority (91%) had some APOE genotyping from this cohort with enriched AD risk, and 41% had at least one APOE4 allele. Approximately half (n = 209) of these scans were also quantified as amyloid and/or tau positive using the methods described inBetthauser et al. (2020). The demographic information of the participant scans is given inTable 2. Note that the number of PET scans (n) includes scans from participants with multiple (i.e., longitudinal) time points and because not every participant scan was labeled as amyloid or tau positive, the total number of scans does not equal the sum of the amyloid and tau groupings.

**Table 2.** Description of UW [F-18]MK6240 imaging cohort.

	A-,T- (n = 142)	A-,T+ (n = 18)	A+,T- (n = 29)	A+,T+ (n = 20)	Total (n = 433)
Female (%)	67%	61%	72%	70%	67%
Age	61.4(6.5)	60.7(4.2)	64.7(5.5)	65.9(4.7)	68.7(7.3)
Weight (kg)	80.9(17.9)	81.5(21.7)	76.5(17.4)	75.0(20.7)	80.4(18.3)
Longitudinal (%)	42%	33%	52%	45%	30%
APOE4+ (%)	37%	22%	83%	45%	41%
Unimpaired (%)	95%	83%	79%	85%	87%
MCI (%)	4%	11%	14%	10%	9%
Dementia (%)	1%	6%	7%	5%	3%

There was a wide range of ECB in the meninges and sinus between participants and scans at our institution. The sinus SUVR ranged from 0.72–3.09 with an average of 1.38(0.34), and the meninges SUVR ranged from 0.57–2.10 with an average of 1.03(0.25) as shown inFigure 5. The meningeal ROI used for this investigation circumscribes the entire brain. It was also observed that the meningeal voxels adjacent to the inferior cerebellar reference region demonstrated a high concordance (Pearson correlation: ρ = 0.98) with the whole meninges, suggesting the global meninges signal was a valid representation for estimating spill-in corrections to the inferior cerebellar reference region.

**Fig. 5.**
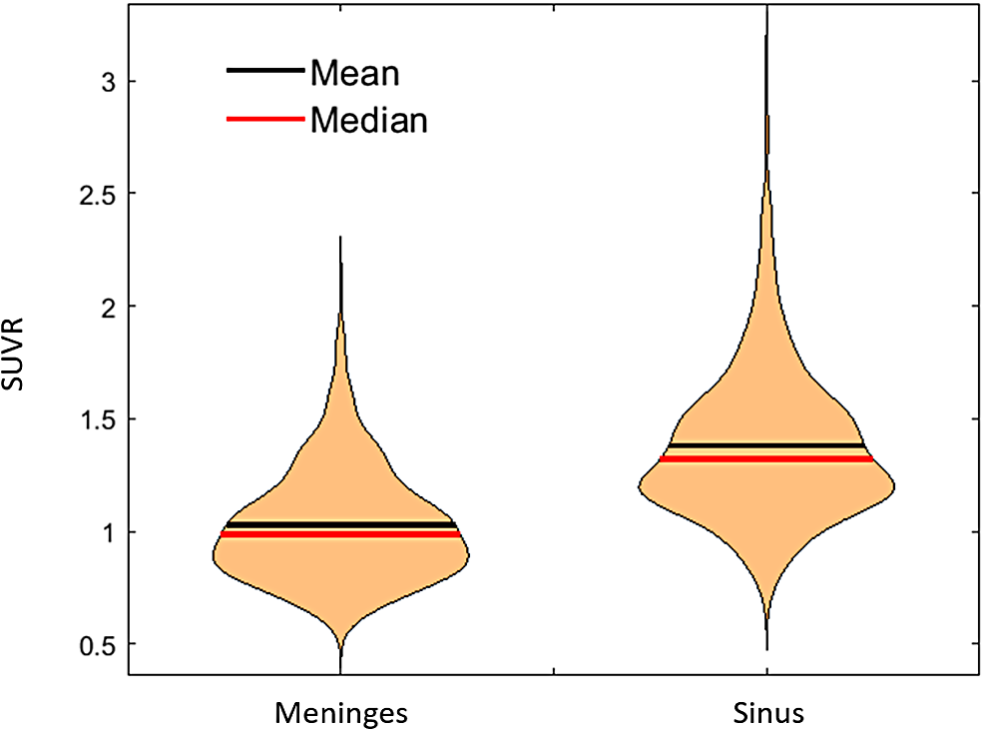
Violin plot showing the distribution of the SUVR in the meninges and sinus of participants in our imaging cohort.

No significant differences in ECB signal between amyloid or tau status were observed (Table 3). There was a significantly higher average SUVR in the meninges and sinus for female and APOE4+ participants; however, there was no significant correlation between ECB and cognitive status or age.

**Table 3.** Differences in ECB between demographic groups.

	Meninges (SUVR)	Sinus (SUVR)	Meninges (p-value)	Sinus (p-value)
Male	0.89(0.16)	1.26(0.26)	p < 0.01	p < 0.01
Female	1.10(0.25)	1.44(0.33)		
APOE4-	0.99(0.23)	1.33(0.29)	p < 0.01	p < 0.01
APOE4+	1.07(0.24)	1.42(0.34)		
A(-)	1.01(0.24)	1.35(0.32)	p = 0.45	p = 0.89
A(+)	0.97(0.21)	1.34(0.42)		
T(-)	0.98(0.23)	1.28(0.23)	p = 0.76	p = 0.33
T(+)	1.00(0.20)	1.28(0.23)		
CN	1.03(0.24)	1.37(0.32)	p = 0.59 p = 0.81	p = 0.26 p = 0.63
MCI	1.05(0.26)	1.43(0.29)		
Dementia	1.01(0.30)	1.33(0.33)		

**Fig. 6.**
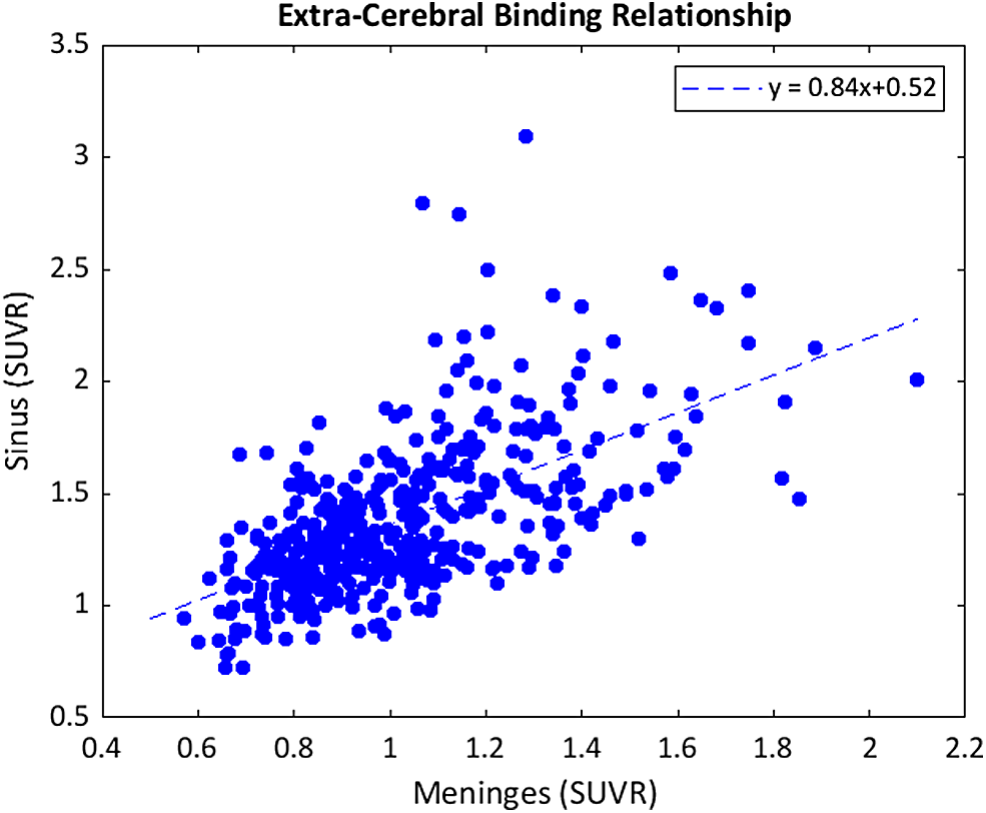
Scatter plot showing the relationship between meninges and sinus SUVR taken from the same participant scan.

A significant linear relationship was observed between the magnitude of PET signal in the meninges and sinus.Figure 6demonstrates this relationship of increasing meninges SUVR with higher sinus SUVR (ρ = 0.61, p = 0.99).

In our ROC curve analysis of the meningeal SUVR compared to visual scoring of meningeal signal from 0–5, we found a maximum Youden’s Index value for an SUVR threshold of 1.07 to be considered Moderate ECB (J = 0.59) and an SUVR of 1.34 for the High classification (J = 0.87). Thus a meningeal SUVR between 0.57 and 1.07 was classified as Low ECB (65% of all participants), between 1.07 and 1.34 had Moderate ECB (24%), and SUVR between 1.34 and 2.10 were classified as High ECB (11%).

### 3.2 Longitudinal comparison of extra-cerebral signal

**Fig. 7.**
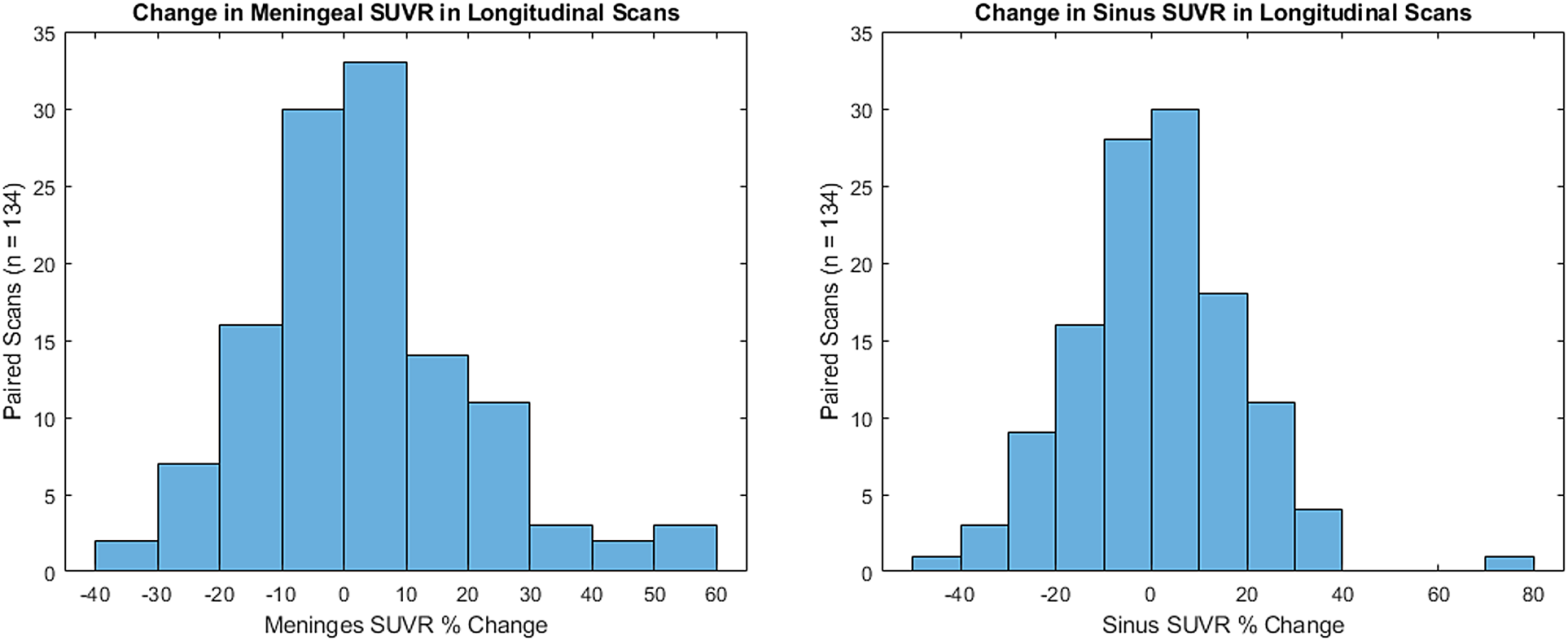
Histograms showing the distribution of change in meningeal (left) and sinus (right) SUVR between scans of the same participant. Bin sizes are increments of 10% change centered about 0.

A total of 129 participants included in the analyses had at least two [F-18]MK6240 scans, including four participants with three timepoints, with an average time of 2.42(0.60) years between scans. Assuming typical 10% test-retest variability in PET outcome measures, these longitudinal studies revealed 49% of participants with greater than ±10% change in the sinus and 44% in the meninges in subsequent scans. On average, a percent difference of just 0.9(17.9)% was observed in sinus SUVR and 1.3(16.4)% in meninges SUVR between scans of the same participant. However, when comparing the absolute difference (i.e., the difference from a change of 0) between scans this increases to an average 13.2(12.2)% for sinus and 12.1(11.2)% for meninges SUVR between scans in our cohort.Figure 7shows a histogram of the distribution of the percent change in the meninges and sinus between scans of the same participants with longitudinal data.

Using the meningeal and sinus ROI to quantify ECB, scans were identified with the lowest sinus and meninges SUVR (0.72 and 0.69), the highest meningeal SUVR (2.10). and the highest sinus SUVR (3.09). These three scans served as the template of our LOW, H_MENINGES and H_SINUS simulation groups described inTable 1. The H_MENINGES case was noted as being particularly interesting due to having a higher meningeal than sinus SUVR, which was uncommon for this cohort.

### 3.3 Validation of [F-18]MK6240 simulations

**Fig. 8.**
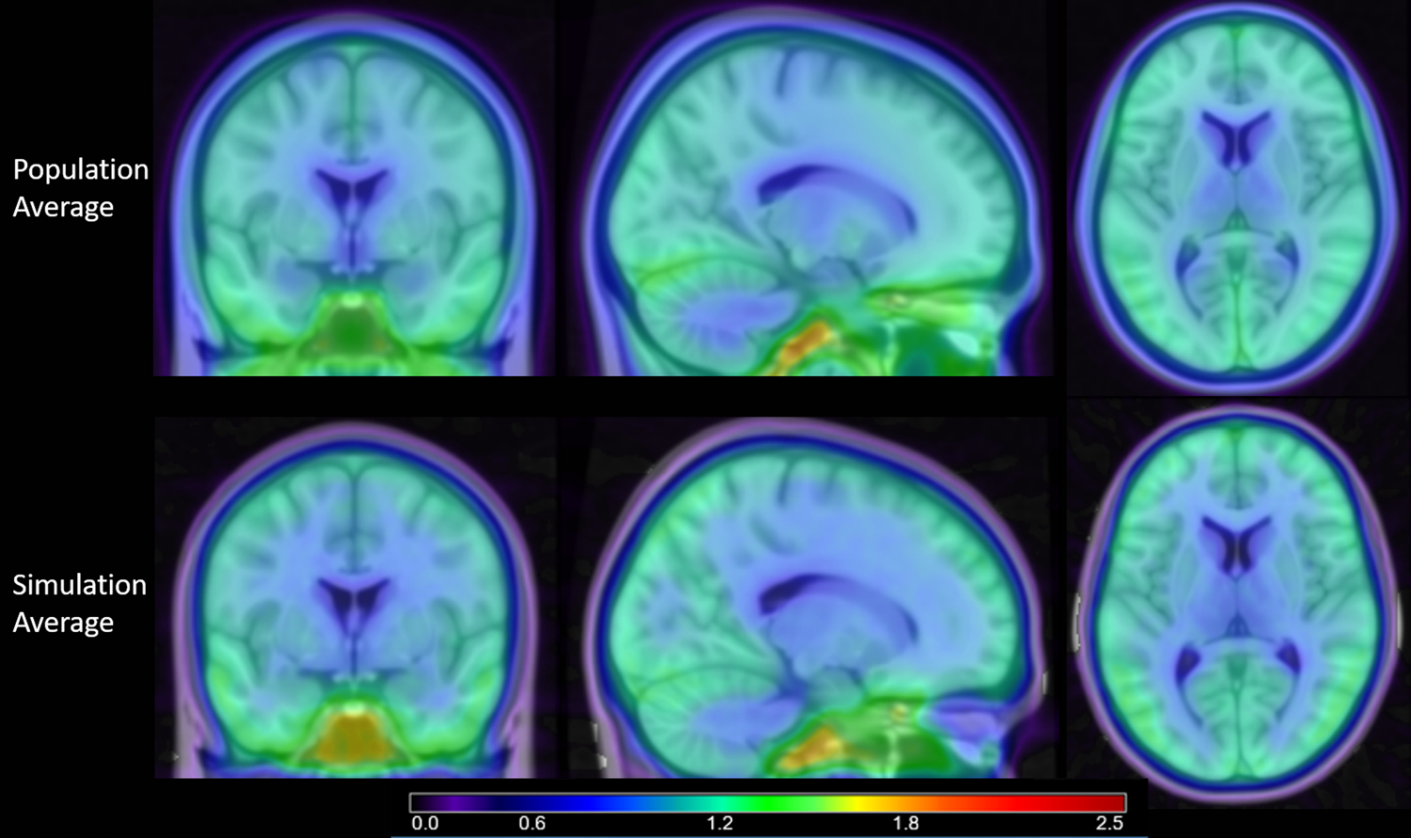
Images comparing the average of the 433 [F-18]MK6240 scans (top) with the average of 100 simulations from the AVG group (bottom).

The population average of the 433 [F-18]MK6240 PET scans used in these analyses and the average of 100 realizations of the AVG group visually have similar radiotracer distributions (Fig. 8). The SUVR of target regions in the mesial temporal lobe near regions of earliest tau accumulation, including the ERC and HC, as well as the IC, a region of relative isolation from ECB effects, are all well within 5–10% of the population average, the test-retest variability typical with PET imaging (Table 4). Note that the coefficients of variation for the population averages are much higher than the simulation group due to including variation in radiotracer distribution and morphology naturally seen between study participants. In the simulated data, these are removed, and the variation is instead driven by PET scanner properties and simulated statistical fluctuations.

**Table 4.** Comparison of average simulation and participant data.

	Meninges (SUVR)	Sinus (SUVR)	ERC (SUVR)	HC (SUVR)	IC (SUVR)
Population	1.05(0.22)	1.41(0.31)	1.03(0.32)	0.90(0.30)	0.98(0.16)
Simulation	1.03(0.01)	1.38(0.02)	1.06(0.04)	0.91(0.03)	0.95(0.02)

### 3.4 Simulated spill-in effects

**Fig. 9.**
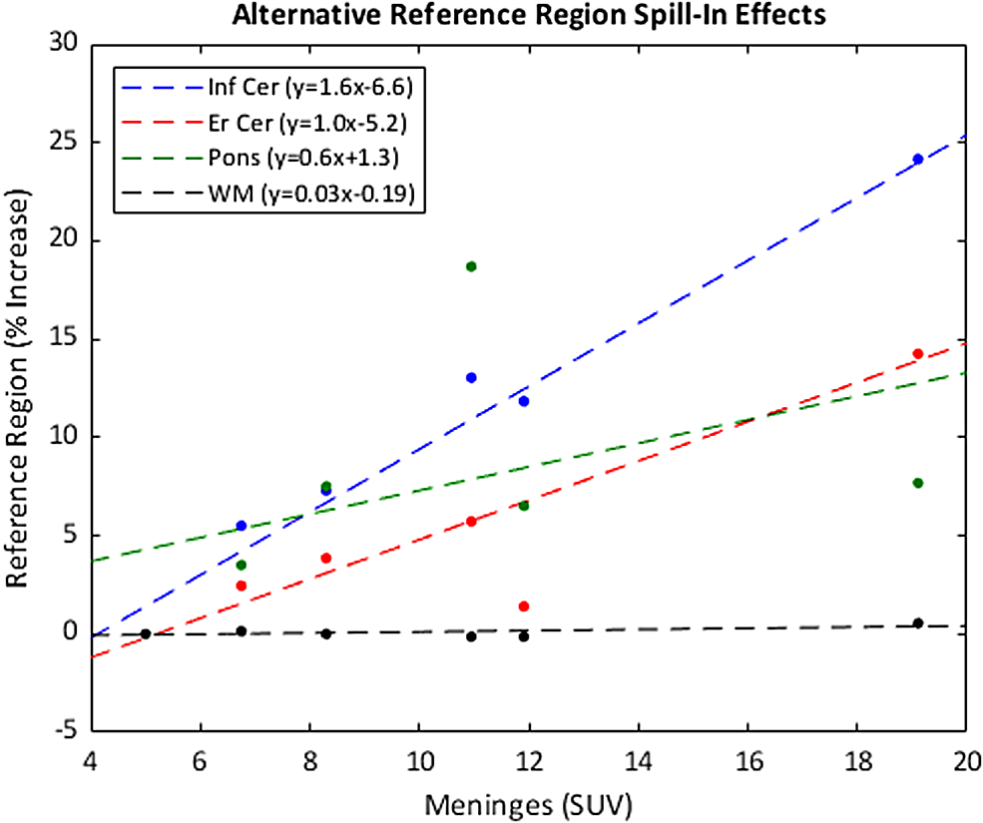
Plots showing the relationship between the ECB signal in the meninges and the influence on potential [F-18]MK6240 reference regions in our simulated data. The cerebral white matter distribution is nearly flat for this range.

Comparing the meninges SUV to those of the alternative reference regions (eroded cerebellar grey matter, cerebral white matter, and the pons), a decreased influence of the meninges compared to the inferior cerebellar grey matter was observed (Fig. 9). The percent increase from the BKG to higher ECB simulations is lower for the eroded inferior cerebellar reference region compared to the inferior cerebellum at all points evaluated and the slope (indicating the relationship between increasing meninges SUV and spill-in signal to the reference region) decreases to 1.0 from 1.6%/SUV for the eroded inferior cerebellar grey matter reference region. While the pons also appears to perform better than the inferior cerebellum due to its decreased slope (0.6%/SUV), it is more directly influenced by ECB signal from the sinus compared to the meninges, as evidenced by the much higher SUV seen in the H_SINUS (SUV_meninges_= 10.95, SUV_sinus_= 29.89) simulation compared to the linear fit. The cerebral white matter reference region is not greatly influenced by increasing meninges or sinus SUV, with the difference between the highest and lowest values (7.34 and 7.29 respectively) having a percent difference of less than 1%.

**Fig. 10.**
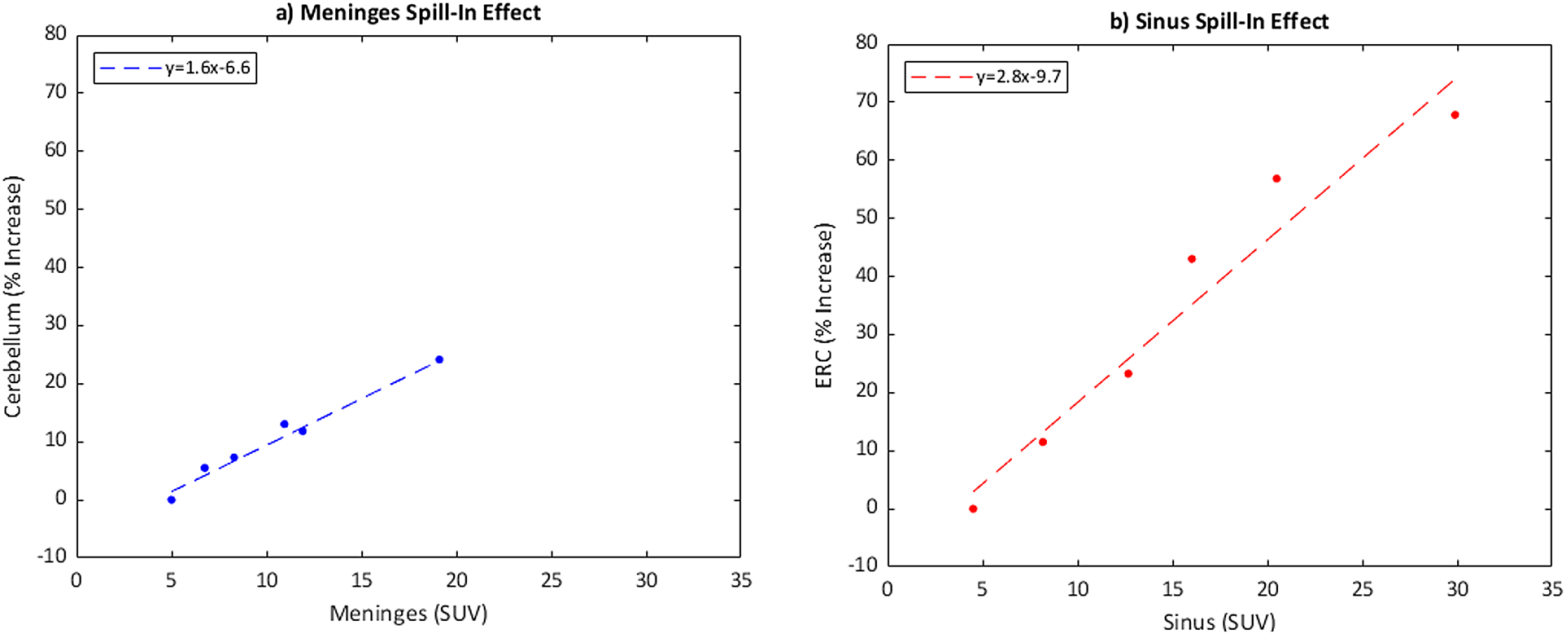
Scatter plots showing how cerebellar SUV increases with higher meningeal signal (a) and ERC SUV increases with the sinus (b).

In these simulations evaluating brain regions typically of highest importance in [F-18]MK6240 PET imaging, as the ECB signal in the meninges and sinus increased, there was a corresponding increase in the average SUV of the inferior cerebellar grey matter and ERC, respectively (Table 5). In the IC, selected as an interior cortical brain region distant from expected ECB effects, there was relatively little change in the average SUV and all groups were within one standard deviation. Plotting meninges SUV against % increase in cerebellar SUV from the BKG simulation (Fig. 10a) demonstrates a strong linear relationship with a Pearson linear correlation coefficient ρ = 0.99. A linear relation between sinus SUV and % increase in ERC SUV (Fig. 10b) also reveals a strong association (ρ = 0.97). Following this relationship, a [F-18]MK6240 signal increase of 99% in the meninges from background yields a greater than 10% increment in the measured cerebellar signal. The ECB influence was even more pronounced with the sinus, with a 52% increase required for an increase of 10% in the neighboring ERC SUV.

**Table 5.** SUV of simulations in target and ECB regions.

ECB level	Meninges (SUV)	Sinus (SUV)	Cerebellum (SUV)	ERC (SUV)	IC (SUV)
BKG	5.00(0.02)	4.51(0.05)	8.36(0.04)	7.74(0.22)	8.15(0.14)
LOW	6.75(0.02)	8.18(0.13)	8.82(0.04)	8.63(0.24)	8.22(0.13)
AVG	8.30(0.02)	12.68(0.09)	8.97(0.03)	9.54(0.18)	8.14(0.21)
HIGH	11.91(0.03)	16.03(0.10)	9.35(0.06)	11.07(0.28)	8.22(0.11)
H_MENINGES	19.11(0.03)	20.49(0.15)	10.38(0.07)	12.14(0.33)	8.23(0.23)
H_SINUS	10.95(0.02)	29.89(0.13)	9.45(0.04)	12.99(0.32)	8.27(0.17)

Using the equation for the linear relationship of these data, a scanner specific correction factor for ECB spill-in to target ROI was generated. The corrected value, SUV’, can be found using the formula for the ERC:

$$SUV\prime_{ERC}=SUV_{ERC}-\left(0.22*SUV_{Sinus}-0.75\right)$$ (1)

and for the cerebellum:

$$SUV\prime_{Cerebellum}=SUV_{Cerebellum}-(0.14*SUV_{Meninges}-0.55).$$ (2)

### 3.5 Detection thresholds for elevated [F-18]MK6240 binding

**Fig. 11.**
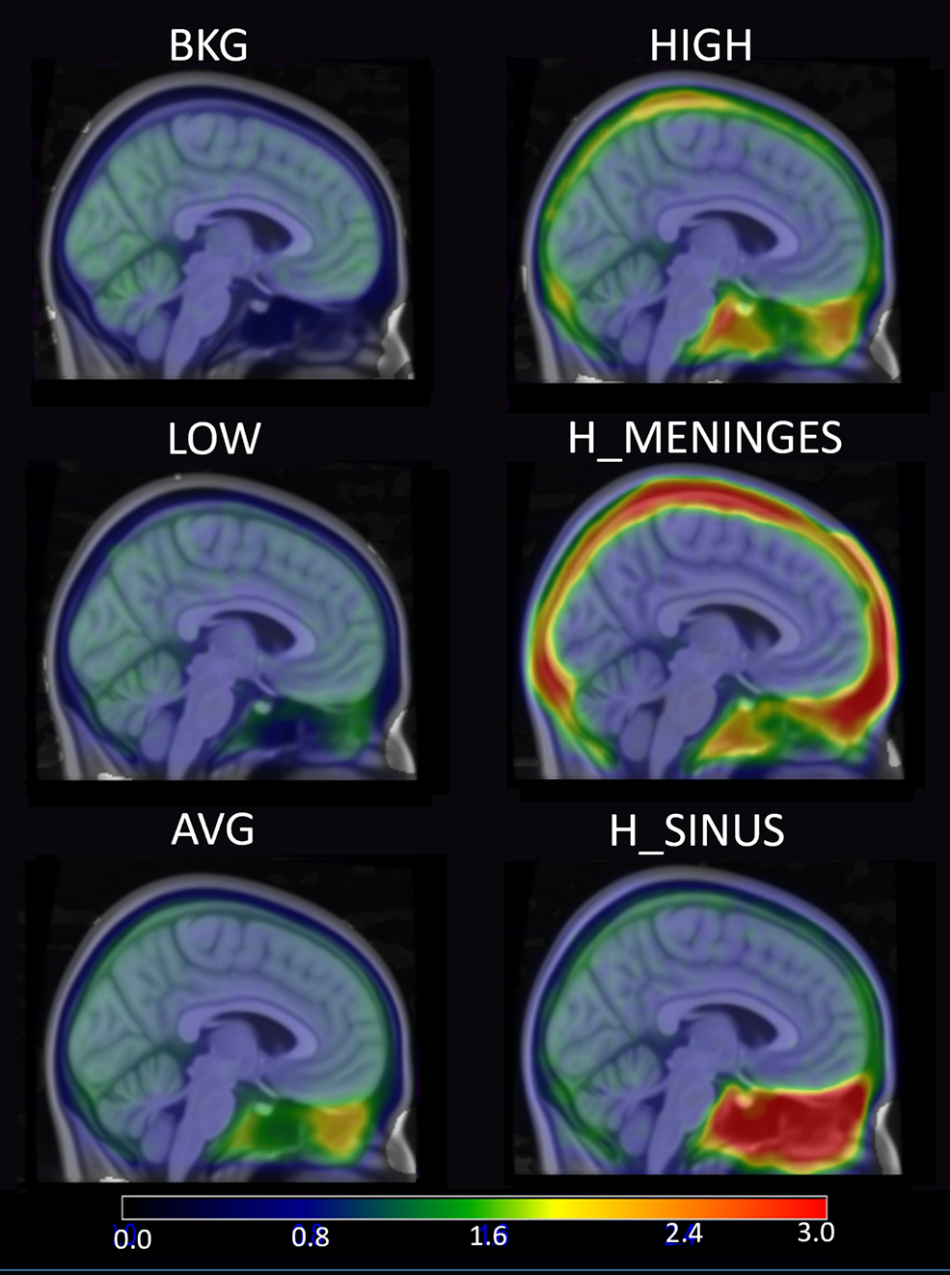
Average of 10 realizations of each tau negative group ordered by increasing sinus SUVR from top to bottom, left to right.

Using the average ECB signal from the AVG group, the results revealed that the RTCC for [F-18]MK6240 in the Braak I region (defined in the high-resolution atlas) required a concentration of 470% greater than the reference region to exceed the established tau positivity threshold of SUVR = 1.27 in the simulated image. The required ERC signal was*reduced*for the HIGH group, with [F-18]MK6240 concentrations decreased to 300% reference for the HIGH group, corresponding to a 30% reduction in tau concentration necessary for quantification when compared to the AVG simulations. Due to the presence of spill-in from the sinus region, the H_SINUS group simulations were identified as tau positive with no specific binding in the Braak I region and an SUVR of 1.38(0.03). For simulations in the BKG and LOW ECB groups, the required radioligand concentration required to be detected as tau positive increased to 560% and 690% greater than reference, respectively. However, despite having the second highest sinus signal (i.e., SUV) of the simulations, the H_MENINGES group also demonstrated an increased input activity concentration of 520% required to reach tau positivity, which is 9% higher than the AVG group. Using the average of the 10 realizations of each simulation group (Fig. 11), the SUV and SUVR of these images are given below inTable 6along with the % difference between the input radioactivity concentration and the AVG cohort.

**Table 6.** Average simulated signal.

ECB level	Input activity vs. AVG (%Δ)	ERC (SUV)	Cerebellum (SUV)	ERC (SUVR)
BKG	+39%	10.90(0.15)	8.35(0.02)	1.31(0.02)
LOW	+16%	11.35(0.18)	8.78(0.05)	1.31(0.02)
AVG	0%	11.58(0.25)	8.99(0.04)	1.31(0.02)
HIGH	-30%	12.27(0.14)	9.38(0.06)	1.32(0.02)
H_MENINGES	+9%	13.81(0.33)	10.39(0.06)	1.33(0.03)
H_SINUS	-82%	12.99(0.32)	9.45(0.04)	1.38(0.03)

### 3.6 Accuracy of correction factors in simulated data

The percent change in SUV across the ECB distribution simulations from the ground truth BKG group in the ERC and cerebellum across the uncorrected images, corrected using the derived SCF (Equations 1and2) and PVC-corrected are given inTable 7ordered by increasing sinus SUV.

**Table 7.** SCF and PVC % difference from BKG simulations.

ECB level	ERC %Δ (uncorrected)	ERC %Δ (SCF)	ERC %Δ (PVC)	Cerebellum %Δ (uncorrected)	Cerebellum %Δ (SCF)	Cerebellum %Δ (PVC)
LOW	11.4%	1.0%	8.6%	5.5%	2.6%	4.6%
AVG	21.3%	0.0%	15.8%	7.3%	1.8%	6.3%
HIGH	42.9%	10.5%	32.1%	11.9%	0.3%	9.4%
H_MENINGES	56.8%	11.7%	48.5%	24.2%	0.6%	19.2%
H_SINUS	67.8%	4.4%	58.2%	13.0%	3.1%	10.8%

Note: %Δ = |Sim – BKG|/BKG.

### 3.7 Application of derived simulation correction factors to participant data

**Fig. 12.**
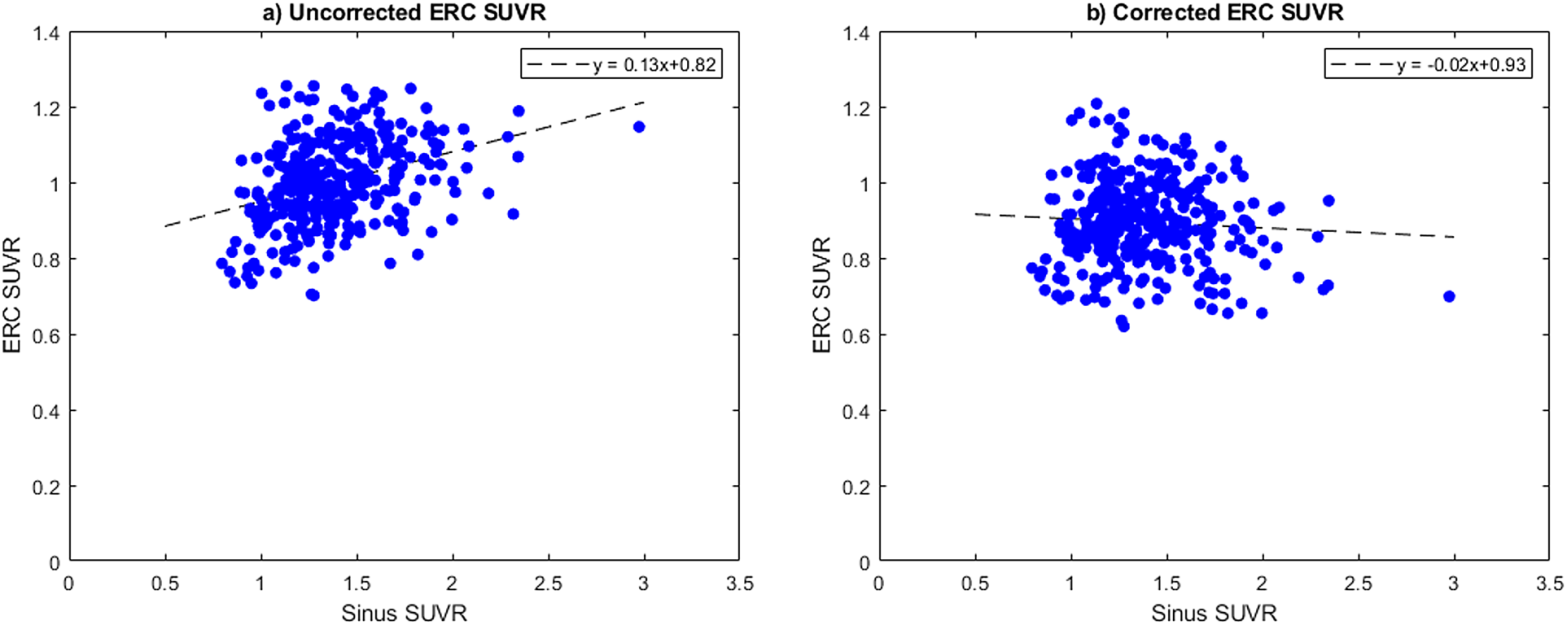
Plots showing the relationship between the sinus and ERC SUVR in our imaging population before (a) and after (b) correction using the SCF derived in this paper.

The SCFs derived in these analyses were applied to the human participant image data acquired on an ECAT HR+ scanner to evaluate the change in the derived SUVR metric. Plotting sinus SUVR against the ERC in our tau negative population, we see a positive correlation between the two regions (Fig. 12a) before correction. Tau negative participant scans were divided into quartiles based on sinus SUVR with the ranges of the quartiles being: Q1[0.80,1.18], Q2[1.18,1.35], Q3[1.35,1.56], and Q4[1.56,297]. Comparing the ERC SUVR of each quartile, we see significant differences between Q1 with Q2 (p = 0.02), Q3 (p < 0.001), and Q4 (p < 0.001), respectively. After applying our SUV corrections to the ERC and inferior cerebellum and calculating a corrected SUVR, the distribution across sinus uptake flattens and the slope of the linear fit decreases from 0.13 to -0.02 (Fig. 12b). The group differences between Q1 with Q2 (p = 0.50), Q3 (p = 0.25), and Q4 (p = 0.53) are also not significant after correction using the SCFs.

## 4 Discussion

[F-18]MK6240 is a widely used and validated PET tracer for quantifying tau pathology in AD progression (Gogola et al., 2022;Hostetler et al., 2016). However, our results in this study demonstrate that the widespread prevalence of ECB in the meninges and sinus in [F-18]MK6240 scans can introduce bias from signal spill-in effects to target and reference regions in clinical research populations. The low levels of neuropathology, and corresponding PET signal, during the early phase of tau aggregation seen in preclinical AD preceding cognitive impairment requires accurate quantitative of [F-18]MK6240 binding to characterize the role of tau during this critical stage of disease progression. This work demonstrates the prevalence of ECB in our large imaging cohorts and isolates the effects of the PET signal spill-in from the meninges and sinus through simulated PET images. These findings offer insight into the influence of ECB signal that can help inform analyses measuring early tau deposition in AD in the presence of confounding signal in the meninges and sinus.

### 4.1 Characterization of imaging cohort

To our knowledge, this work quantifying ECB in the University of Wisconsin cohorts is the largest investigation of [F-18]MK6240 signal in the meninges and sinus to date. The observed presence and spatial extent of [F-18]MK6240 ECB using this cohort of 300 participants with 433 PET scans are consistent with other investigations of ECB using [F-18]MK6240 (Aguero et al., 2019;Baker et al., 2021;Harrison et al., 2023;Pascoal et al., 2018;Salinas et al., 2020;Smith et al., 2021;Tissot et al., 2023;Vanderlinden et al., 2022). However, a physiological understanding of the enhanced [F-18]MK6240 meningeal and sinus signal is not as well understood. [F-18]MK6240 has been shown to bind to melanin containing cells, including extracutaneous meningeal melanocytes (Aguero et al., 2019). It has been previously reported that [F-18]MK6240 radio-metabolites do not enter the brain in significant amounts (Hostetler et al., 2016;Pascoal et al., 2018). However, accumulation of [F-18]MK6240 metabolites in the sinus cavities may contribute to the PET signal in this region, which could be related to the presence of ECB signal in [F-18]MK6240 PET images.

Our results demonstrate that ECB profiles vary between participants, and to a lesser extent between scans of the same participant. Overall, the ECB patterns follow a consistent general uptake profile with the highest activity in the proximity of the ventral frontal lobe, posterior cerebellum, dorsal parietal and medial temporal lobes. The longitudinal investigations reveal an average absolute percent increase of 13.2(12.2)% in the sinus and 12.1(11.2)% in the meninges, which is higher than the values previously identified inVanderlinden et al. (2022), which examined longitudinal changes in ECB for both CN and MCI participants. There are several differences in our methods, the most notable being the definition of the ECB region. In this referenced work, the ECB region was not separated into the meninges and sinus and instead used a Freesurfer skull parcellation that includes bone and the meninges. Our analysis was focused more on the regions of highest uptake in the meninges and sinus, which can explain the difference in percent change between scans, but the similarities seen in variability. At this time, we offer no physiological explanation for the high variability seen between longitudinal scans of the same participant, which was observed in both quantitative and qualitative-reader measures. As such, we must acknowledge potential uncertainties introduced by image processing methods, primarily because the images were processed in standard space (and not native space). In regions outside the brain like the meninges and sinus, it is acknowledged that small inconsistencies in registration and segmentation between scans of the same participant could increase the variance of our ECB measures. While our analysis pipeline is applied consistently to each scan and differences in registration are typically small, the pipeline processes images cross-sectionally, which has a higher intrasubject variability than longitudinal image processing, and regions outside the cerebrum including the meninges and sinus are more vulnerable to registration differences due to the lack of structural MRI information.

A total of 152 participant scans included in this study (over 30% of the 433 total scans) had meningeal signal classified as either Moderate or High that could potentially bias the SUVR outcomes. While our classification system did not include the signal from the sinus, visual inspection of this region revealed signal contamination to the entorhinal cortex in many of the images. Overall, a high prevalence of ECB was observed in this cohort, particularly in regions that could adversely affect the accuracy of the measured [F-18]MK6240 SUVR. While visual reads of the SUVR images can be used to identify the presence and gauge the magnitude of ECB, challenges exist with accurately assessing their PET signal contribution to brain regions with early tau accumulation, such as the entorhinal cortex. Further, the performance of algorithms to correct for signal spill-in or -out cannot be fully validated without precise knowledge of the source radiotracer distribution. Understanding the influence of the ECB on the quantitative assessment of [F-18]MK6240 binding motivated this investigation for creating the simulation image data.

While the participants included in this study were restricted to only those acquired using a single PET scanner (Siemens ECAT HR+) at our center, our standardized [F-18]MK6240 pipeline does include a 6 mm Gaussian smoothing step to match image resolution with other PET scanner platforms at our site, as well as those from multi-center collaborations. We acknowledge that introducing additional smoothing to the reconstructed image does increase the influence of ECB but feel the inclusion of this processing step will make our findings more generalizable across a wide range of PET scanner models. It should also be noted that for these studies, an imaging acquisition window of 70–90 minutes post-injection of [F-18]MK-6240 was used rather than a commonly used later window of 90–110 minutes. Our past findings have shown high correlation between ground-truth DVR values and the 70–90 minute window for SUVR (validated inBetthauser et al. (2019))and that the signal in target regions reaches pseudo-equilibrium by 70 minutes. Further, an increasing PET signal in the meninges and sinus relative to the inferior cerebellar reference region was observed with later acquisition times, making the target region SUVR more vulnerable to bias from the ECB signal when analyzing later time frames.

### 4.2 Simulated images

The simulated [F-18]MK6240 image data were modeled using the large cohort of participant data at our site, all undergoing standardized acquisition and preprocessing. This offers the advantage of carefully examining the effects of ECB at a population level separate from issues of data harmonization between PET scanners or imaging sites. Furthermore, the use of simulated images permits the removal of many sources of variance in PET imaging that could potentially obfuscate the influence of ECB in target regions, including differences in radiotracer metabolism and distribution between participants. The simulation groups were based upon cohort averages and case examples with unique ECB binding patterns to examine a full range of potential clinical research scans. These case examples included the LOW simulation cohort, representing the lowest amount of ECB observed in our human studies, and H_SINUS and H_MENINGES, demonstrating the highest sinus and meningeal signal. Simulations of more typical scans (including AVG and HIGH) closely follow the most prevalent ECB profiles of [F-18]MK6240 uptake with the results from these cohorts being most representative of anticipated [F-18]MK6240 human scans.

For the validation of the SORTEO simulation tool, the average of the 100 realizations in the baseline AVG group were visually similar to the population average of 433 scans with clear delineation of grey matter, white matter, and CSF in the ventricles. Edges between brain regions demonstrated greater sharpness (i.e., contrast) in the simulated images because they are based on a binarized emission map between distinct brain regions which may be more realistically represented as a continuous distribution of radioligand in human scans. However, the overall pattern of [F-18]MK6240 distribution is maintained and ECB within the sinus and meninges are also closely preserved in the simulations. Quantitatively, the SUVR of the average of the simulated images are in agreement with the SUVR from the human population average for target tau regions in the medial temporal lobe. The simulation average SUVR in the ERC, HC, and IC are all within the expected 5–10% variability of [F-18]MK6240 PET imaging compared to the human population average, providing confidence that the SORTEO simulated images can be used to accurately model early tau accumulation analysis in the medial temporal lobe.

It should be noted that the standard errors of the simulations are lower than that of the human data by an order of magnitude. This was expected because the simulations are derived from a single distribution of [F-18]MK6240 in standard space, with the realizations representing the variation due to statistical noise from the radioactive decay and detection processes for a specific PET scanner model. Thus, variance due to differences in metabolism and morphology between participants, registration errors, and differing radiotracer distributions are not present in the simulated cohorts. The exclusion of variance related to these sources permits the focused examination on ECB and the effects of changes in meningeal and sinus signal that otherwise could not be separated from other sources of variability.

In the simulations, there are several brain regions where there is lower PET signal than observed in the human scans. This mismatch is most pronounced near the edge of the frontal lobe and could be remedied by modeling PET signal from the optic nerve. However, because this region is distal from the target and reference regions, it was not included in our model in order to reduce the computer processing time of each simulation. Similarly, areas on the inferior edge of the cerebrum, which is near the border of our emission map, showed evidence of lower estimated PET signal. Because this region is near the limits of the PET scanner field-of-view, no effort was made to address this mismatch. It should be noted that if accurate representation of the PET signal in the orbitofrontal cortex is required, then additional brain regions (e.g., optic nerve) should be included for generating the simulation images.

The occipital edge of the brain also has slightly higher activity concentration in our simulations. This is one of the most prevalent brain regions showing meninges binding. While not consequential for interpreting [F-18]MK6240 scans for AD-related investigations, the resulting PET signal for the occipital lobe may be slightly overestimated in the simulated images. This combined with the spill-in from our simulated ECB profile may cause a double counting effect where the derived RTCCs already account for meningeal spill-in, but even more signal is added in the simulations from the meninges. Other areas of the brain have more heterogeneous ECB, so we would not expect to see population-wide increases in the medial temporal lobe and inferior cerebellar grey matter RTCCs due to spill-in effects.

While in our validation of PET SORTEO there was agreement between our simulated data and the participant scans along the superior edge of the cerebellum, we acknowledge there is also a region of meningeal off-target binding between the temporal lobe and the cerebellum that is not covered by our meninges mask. This section of the meninges is included in the temporal and cerebellar ROIs in our simulations and is thus kept constant between simulation cohorts. The effects of spill-in to the reference region are mitigated by using only the inferior cerebellum; however, there is the potential for target region spill-in to the neighboring regions in the temporal cortex. Much of the spill-in signal in this region is dominated by contributions from the sinus and because a strong correlation between the sinus and meninges uptake was observed, potential meninges spill-in to the proximal entorhinal and inferior temporal cortex would be proportionally minor. However, additional corrections would be necessary if a mismatch between sinus and meninges uptake exists.

It is also acknowledged that while the simulations and participant data have the same resolution in common space, the post-processing of these image sets are not identical. The simulated images were not smoothed after reconstruction, while there was a 6 mm Gaussian smoothing kernel applied to the participant data before warping into standard space. Our simulated data used RTCCs based on (and intended to recreate) the radiotracer distribution of the already smoothed human participant images in common space. Thus, no further post-processing smoothing was applied to the simulated data already in common space. While our validation of the simulation tool and the derived correction factors do show close agreement with our participant data, in recreating our results at different sites and different scanners our methods could be modified by using unsmoothed data as the source of the simulation RTCCs.

### 4.3 Detection thresholds of [F-18]MK6240

SUVR thresholds are often used to establish a cutpoint for categorizing an image as tau “positive” or “negative.” The spill-in signal from the sinus region produces an elevated PET signal in the mesial temporal lobe, potentially masking the signal of [F-18]MK6240 that is specifically bound to tau pathology in this region. Conversely, the increasing spill-in signal to the cerebellar reference region decreases the global and regional SUVR of the image. The use of simulated data allows us to carefully characterize the amount of signal contamination present and the effects of this contamination on the level of tau binding required to reach the positivity cutpoint. To address the issue of tau positive detectability in the presence of ECB, we systematically altered the model input [F-18]MK6240 radioactivity signal in the target region, in this case the NFT distribution seen in Braak I tauopathy, in the presence of ECB.

In these simulations, the detection thresholds for the model input activity of [F-18]MK6240 were highly dependent on the ECB spatial pattern. Simulations with below average sinus binding (BKG, LOW) required input [F-18]MK6240 concentrations 39% and 16% higher, respectively, representing a higher concentration of tau pathology in the Braak I region to be classified tau positive. Those with above average sinus signal (HIGH, H_SINUS) required a lower concentration. In the H_MENINGES simulations, however, the effect of the meninges spill-in to the cerebellar reference region required an [F-18]MK6240 activity concentration 9% higher in the target region than the AVG simulations to be classified as tau positive.

This counterintuitive finding can be explained by the role of the inferior cerebellar reference region in calculating SUVR in [F-18]MK6240 images. The inferior cerebellar grey matter is used as a reference region for [F-18]MK6240 due to its lack of significant tau pathology and relative isolation from brain regions with potentially high signal such as the occipital lobe. When this reference region produces an inflated signal due to spill-in from the meninges, there is a corresponding decrease in the global SUVR. As spill-in to the reference region increases, the SUVR in interior regions such as the IC are increasingly underestimated in scans with high meningeal uptake just as radioactivity from the sinus can cause neighboring regions in the temporal cortex to be overestimated in [F-18]MK6240 analysis. The competing influences of the meninges on the cerebellum signal and the sinus on the ERC signal reveals that higher sinus SUVR does not necessarily correlate with a lower input activity threshold. Spill-in from the meninges into the cerebellum drives down the global SUVR; however, peripheral regions such as the ERC also see spill-in from the sinus that can offset this global SUVR decrease, complicating the ability to use a uniform cut point for tau positivity in a large cohort study. These findings mirror the conclusions ofHarrison et al. (2023), which proposed that the spill-in effects to the meninges and target regions can offset each other in SUVR analysis. However, due to the differing rates of spill-in contributions from the meninges and sinus and the heterogeneity of ECB seen in our imaging cohort, this effect is not consistently reliable, making the use of correction factors more effective for the removal of ECB signal bias. For participants with very high ECB patterning, especially when primarily located in the sinus, PET measures such as SUVR would benefit from a visual read by a trained imaging specialist to identify outliers that may require extra analysis to avoid biasing outcomes.

These simulations represent a specific time point in tau progression, with a focus on the early tau aggregation in the ERC as observed in PET investigations. No attempt was made to source the simulated PET signal to a tissue-based assay of tau concentration (e.g., B_max_). More complex atlas representations with disease progression of tau concentration and spatial extent will be needed to fully capture the influences of ECB on the measured [F-18]MK6240 SUVR throughout the AD continuum. The established tau positivity threshold of an SUVR of 1.27 in the ERC is also based on a large population with extensive variation in ECB and AD neuropathology. Our simulations demonstrate that this threshold is dependent on the extent ECB. Although a higher (model-input) concentration of radiotracer in the Braak I region is required to reach the tau positivity threshold in the BKG and LOW ECB simulations, it should be noted that a lower threshold could be established in this region using a cohort restricted to low ECB. Correcting the SUVR outcome for ECB will improve the precision of the threshold for characterizing early-stage AD neuropathology for research investigations.

### 4.4 Effects of [F-18]MK6240 signal spill-in on target regions

The PET simulations revealed a substantial measured signal bias introduced by the ECB into the measured radiotracer concentration in AD-related target and reference regions used to calculate SUVR outcomes. The images demonstrated an increase in signal in both the ERC and inferior cerebellar reference regions with increasing ECB, although substantially lower in magnitude than the local PET signal in the ECB regions. As the radioactivity in the sinus increased, there was a greater spill-in to the ERC and a resultant higher SUV and similarly increases to meningeal activity influenced a higher signal in the cerebellum. This is reflected in the linear relationship between the ECB regions neighboring brain regions, as seen inFigure 10. There is a strong linear correlation between both cerebellum and ERC regions; however, the slope of the sinus spill-in effect is nearly double that sourced from the meninges. The IC was selected to illustrate the absence of spill-in signal from the ECB in distal regions, revealing a less than 2% difference in measured signal between all levels of ECB uptake, which is well within one standard deviation. Comparing the inferior cerebellar grey matter to alternative reference regions, a smaller impact of meningeal signal was also observed. In particular, the eroded cerebral white matter reference region performed well and demonstrated no increasing signal with a higher meninges SUV, thus serving as a suitable candidate for identifying a reference region that is less dependent on ECB signal variability.

Despite the observation of some heterogeneity of ECB patterning seen in our human scans, a strong correlation was found between its presence in the meninges and sinus regions. The simulations were then used to demonstrate a consistent level of related spill-in to the brain regions of cerebellum and ERC seen throughout the ECB spectrum. These highly correlated patterns of ECB were exploited to introduce the use of signal correction based upon a simple linear relationship derived from the simulated images. Both the proposed SCF and M-G PVC methods resulted in reduced spill-in effects in all simulated cohorts. In the cerebellum, the SCF method performed well and was within 5% of the true value of the BKG simulations for every simulation cohort. While M-G PVC also reduced the differences between the cohorts, it performed poorly in the simulation groups with the highest meningeal binding, demonstrating a 19% difference in cerebellar SUV between the H_MENINGES and BKG (i.e., ground truth) groups.

Although the SCF for sinus spill-over into the ERC was not as accurate as corrections for the meningeal region, the results for the ERC-recovered signal were reasonable, being within ~10% of the BKG group ground truth value. As with the cerebellum, M-G PVC also reduced the measured signal difference compared to the uncorrected images but did not perform as well with higher ECB profiles. In the H_SINUS group, there was a 58.2% difference from the ground truth ERC SUV following M-G PVC, greatly elevating the signal of this target region for early AD analysis. The SCF method outperformed M-G PVC in both the ERC and cerebellar regions, and this difference became more pronounced with higher sinus and meninges signal. While M-G PVC has the advantage of being easily applied to the entire brain, this method could not recover accurate [F-18]MK6240 PET signals in these critical regions in the presence of ECB. It must be noted that the derived linear corrections reported here are dependent on the spatial resolution of the PET scanner, requiring this relationship to be investigated for other scanners and reconstruction methods. However, it is anticipated that a similar correction could be readily derived for other acquisition and reconstruction configurations. The PET SORTEO platform includes several preprogrammed PET scanner configurations and additional customization is also possible for sites using different PET scanner model. For sites with an existing population of [F-18]MK6240 images, representative scans could be converted into emission maps for simulations to derive scanner-specific correction factors as well.

The correction factors derived for these analyses used images from all participants; however, previous work has shown that there are significant differences between ECB in men and women (Salinas et al., 2020;Smith et al., 2021) as replicated in our imaging cohort (Table 3), and a majority (67%) of our participants in this study were women. While the SCF reported in these analyses was developed for applications to the general imaging population, we must acknowledge that sex-specific imaging corrections may also warrant investigation.

## 5 Conclusions

Our study demonstrates the varying effects that ECB can have on [F-18]MK6240 PET SUVR during the early emergence of tau neuropathology. Radiotracer concentrations in the meninges and sinus are heterogeneous between subjects, and even among different scans from the same subject. Spill-in effects from ECB into the target and reference regions have opposing effects on the SUVR image, and simulation studies were used to quantitate the magnitude of signal contamination and the influence on the measured SUVR outcome. With the emergence of promising new therapies for the treatment and prevention of AD, accurate tracking of changes in PET-measured tau pathology with [F-18]MK6240 will be crucial to assess disease progression and treatment response. Accounting for the presence of ECB with [F-18]MK6240 will be needed to improve the precision of measured tau burden.

## Data Availability

PET imaging data from the University of Wisconsin—Madison Alzheimer’s Disease Research Center can be requested using an online application process (https://www.adrc.wisc.edu/apply-resources). The PET SORTEO simulator is currently available through the Centre d’Exploration et de Recherche Médicale par Emission de Positons (CERMEP) website (https://www.cermep.fr/sorteo/). Any additional data can be made available upon request.
